# Axillary basal cell carcinoma: A retrospective review of 57 cases

**DOI:** 10.1016/j.jdin.2026.08.011

**Published:** 2026-09-04

**Authors:** Julia Giroski, Andrea Berger, Patrick Kupiec, Eric Hossler

**Affiliations:** a Geisinger Commonwealth School of Medicine, Scranton, Pennsylvania; b Department of Population Health Sciences, Geisinger Medical Center, Danville, Pennsylvania; c Dermatology Associates of Rochester, Rochester, New York; d Department of Dermatology, Geisinger Medical Center, Danville, Pennsylvania

**Keywords:** axilla, basal cell carcinoma, epidemiology, retrospective studies, skin neoplasms, sun-protected sites

*To the Editor*: Basal cell carcinoma (BCC) predominantly arises on chronically sun-exposed skin, yet rare cases occur at sun-protected sites. Among these, the axilla merits specific attention for several reasons. First, the axilla is a rarely occluded, hair-bearing intertriginous site with a high density of apocrine glands and a distinct microbiome, features that distinguish it from other sun-protected locations, such as the anogenital region. Second, axillary lesions pose a particular diagnostic challenge because they mimic common benign axillary pathology, including cysts, skin tags, and melanocytic neoplasms. Third, recent genomic studies of BCCs arising at sun-protected sites have demonstrated recurrent PTCH1 alterations without ultraviolet mutational signatures, supporting ultraviolet-independent Hedgehog pathway activation as a driver of tumorigenesis at these locations.^1^^,^^2^ Whether the axillary microenvironment contributes to this alternative mutagenic mechanism remains unknown. We therefore retrospectively reviewed biopsy-proven axillary BCCs diagnosed at our tertiary referral center over a 15-year period.

Fifty-seven axillary BCCs were identified in 56 patients (Table I). Median age was 69 years, and 57.1% were men. Twenty-three patients (41.1%) had a history of BCC, whereas only 1 patient each had immunosuppression, prior axillary radiation, or Gorlin syndrome. Tumors were evenly distributed between the right and left axillae, with a median clinical diameter of 10 mm (Table II). Most lesions demonstrated nodular or superficial histology; only 1 infiltrative BCC was identified.

**Table I:** Patient characteristics

Total patients	56
Gender, *n* (%)	
Male	32 (57.1)
Female	24 (42.9)
Age at biopsy	
Median (IQR)	69 (62-74)
Minimum, maximum	4-91
Female age at biopsy	
Median (IQR)	69 (58-74)
Minimum, maximum	4-87
Male age at biopsy	
Median (IQR)	73 (66-75)
Minimum, maximum	41-91
Race, *n* (%)	
White	51 (91.1)
Unknown	5 (8.9)
History of BCC, *n* (%)	
No	20 (35.7)
Yes	23 (41.1)
Unknown	13 (23.2)
History of other skin cancer, *n* (%)	
No	33 (58.9)
Yes	10 (17.9)
Unknown	13 (23.2)
Gorlin or other BCC syndrome, *n* (%)	
No	46 (82.1)
Yes	1 (1.8)
Unknown	9 (16.1)
Immunosuppression, *n* (%)	
No	46 (82.1)
Yes	1 (1.8)
Unknown	9 (16.1)
Tanning bed use, *n* (%)	
No	2 (3.6)
Yes	1 (1.8)
Unknown	53 (94.6)
Family history of NMSC, *n* (%)	
No	17 (30.4)
Yes	5 (8.9)
Unknown	34 (60.7)

*IQR*, Interquartile range; *NMSC*, nonmelanoma skin cancer.

**Table II:** Axillary BCC characteristics

Total confirmed biopsies	57
Aggressive subtype present, *n* (%)	
No	56 (98.2)
Yes	1 (1.8)
Pigmented, *n* (%)	
No	35 (61.4)
Yes	12 (21.1)
Unknown	10 (17.5)
Solar elastosis present, *n* (%)	
No	55 (96.5)
Unknown	2 (3.5)
Margins involved, *n* (%)	
No	9 (15.8)
Yes	48 (84.2)
Laterality, *n* (%)	
Left	29 (50.9)
Right	28 (49.1)
Clinical size (mm)	
Median (IQR)	10 (6-15)
Missing	21
Biopsy type, *n* (%)	
Excision	5 (8.8)
Punch	1 (1.8)
Shave	51 (89.5)
Biopsy by dermatology, *n* (%)	
No	8 (14.0)
Yes	48 (84.2)
Unknown	1 (1.8)
Clinical DDX, *n* (%)	
No	18 (31.6)
Yes	39 (68.4)
Length of time lesion present, *n* (%)	
Less than a year	10 (17.5)
A year or more	11 (19.3)
Unknown duration	36 (63.2)
Definitive treatment, *n* (%)	
Curettage	23 (40.4)
Excision	10 (17.5)
Mohs micrographic surgery	10 (17.5)
None	8 (14.0)
Unknown	6 (10.5)

*IQR*, Interquartile range.

Clinically, 35.1% of tumors were asymptomatic, and 22.8% were discovered incidentally during examination. BCC was included in the clinical differential diagnosis in 68.4% of cases, although pigmented lesions were frequently mistaken for melanocytic neoplasms. Curettage, excision, and Mohs micrographic surgery were the most common treatments.

To our knowledge, our series of 57 tumors represents one of the largest single-institution cohorts of axillary BCCs reported to date.^3^^,^^4^ Consistent with prior reports, axillary BCCs in our cohort were predominantly nonaggressive histologic subtypes.^3^, ^4^, ^5^ The frequent absence of symptoms and high rate of incidental discovery underscore the importance of complete skin examinations that include intertriginous and sun-protected areas. Clinicians should maintain suspicion for BCC when evaluating persistent axillary lesions, even in patients without classic risk factors. Future molecular characterization of axillary BCCs may clarify whether the rare microenvironment of this site contributes to ultraviolet-independent pathways of tumorigenesis.

### Declaration of generative AI and AI-assisted technologies in the writing process

During the preparation of this work, the authors used ChatGPT to proofread the manuscript for grammatical and spelling errors and to increase the clarity of sentence structure. After using this tool/service, the authors reviewed and edited the content as needed and take full responsibility for the content of the published article.

## Conflicts of interest

None disclosed.

## References

[1] Draper E., Li Y.Y., Mahadevan N.R., Laga A.C., Hanna J., Russell-Goldman E. (2025). Clinicopathologic and molecular characterization of basal cell carcinoma arising at sun-protected sites. Am J Surg Pathol.

[2] Dousset L., Yurchenko A.A., Chanal J. (2025). Genomic and clinical characterization of anogenital basal cell carcinoma: a retrospective and prospective cohort study. J Am Acad Dermatol.

[3] Betti R., Crosti C., Moneghini L., Crespi E., Menni S. (2011). Axillary basal cell carcinoma: additional 25 patients and considerations. J Eur Acad Dermatol Venereol.

[4] LeSueur B.W., DiCaudo D.J., Connolly S.M. (2003). Axillary basal cell carcinoma. Dermatol Surg.

[5] Cohen P.R. (2014). Basal cell carcinoma of the axilla: review of the world literature. Am J Clin Dermatol.

